# Effectiveness of a transdiagnostic internet-based protocol for the treatment of emotional disorders versus treatment as usual in specialized care: study protocol for a randomized controlled trial

**DOI:** 10.1186/s13063-015-1024-3

**Published:** 2015-10-31

**Authors:** Alberto González-Robles, Azucena García-Palacios, Rosa Baños, Antonio Riera, Ginés Llorca, Francisco Traver, Gonzalo Haro, Vicente Palop, Guillem Lera, José Enrique Romeu, Cristina Botella

**Affiliations:** University Jaume I, Research Building II, Office NB2128DD, Vicente Sos Baynat Avenue, Castellon, 12071 Spain; Universidad de Valencia, Valencia, Spain; CIBER Fisiopatología Obesidad y Nutrición (CIBERObn), Instituto Salud Carlos III, Madrid, Spain; Psychiatry Service, Consorcio Hospitalario Provincial de Castellón, Castellon, Spain; School of Medicine, Universidad CEU Cardenal Herrera, Castellon, Spain; Psychiatry Service, Departamento de Salud de la Ribera, Hospital Universitario de La Ribera, Valencia, Spain

**Keywords:** Transdiagnostic, Internet, Randomized controlled trial, Emotional disorders, Depression, Anxiety, Computer-delivered psychotherapy, Neuroticism/behavioral inhibition, Extraversion/behavioral activation

## Abstract

**Background:**

Emotional disorders (depression and anxiety disorders) are highly prevalent mental health problems. Although evidence showing the effectiveness of disorder-specific treatments exists, high comorbidity rates among emotional disorders limit the utility of these protocols. This has led some researchers to focus their interest on transdiagnostic interventions, a treatment perspective that might be more widely effective across these disorders. Also, the current way of delivering treatments makes it difficult provide assistance to all of the population in need. The use of the Internet in the delivery of evidence-based treatments may help to disseminate treatments among the population. In this study, we aim to test the effectiveness of EmotionRegulation, a new transdiagnostic Internet-based protocol for unipolar mood disorders, five anxiety disorders (panic disorder, agoraphobia, social anxiety disorder, generalized anxiety disorder and anxiety disorder not otherwise specified), and obsessive-compulsive disorder in comparison to treatment as usual as provided in Spanish public specialized mental health care. We will also study its potential impact on basic temperament dimensions (neuroticism/behavioral inhibition and extraversion/behavioral activation). Expectations and opinions of patients about this protocol will also be studied.

**Methods/Design:**

The study is a randomized controlled trial. 200 participants recruited in specialized care will be allocated to one of two treatment conditions: a) EmotionRegulation or b) treatment as usual. Primary outcome measures will be the BAI and the BDI-II. Secondary outcomes will include a specific measure of the principal disorder, and measures of neuroticism/behavioral inhibition and extraversion/behavioral activation. Patients will be assessed at baseline, post-treatment, and 3- and 12-month follow-ups. Intention to treat and per protocol analyses will be performed.

**Discussion:**

Although the effectiveness of face-to-face transdiagnostic protocols has been investigated in previous studies, the number of published transdiagnostic Internet-based programs is still quite low. To our knowledge, this is the first randomized controlled trial studying the effectiveness of a transdiagnostic Internet-based treatment for several emotional disorders in public specialized care. Combining both a transdiagnostic approach with an Internet-based therapy format may help to decrease the burden of mental disorders, reducing the difficulties associated with disorder-specific treatments and facilitating access to people in need of treatment. Strengths and limitations are discussed.

**Trial registration:**

ClinicalTrials.gov NCT02345668. Registered 27 July 2015.

## Background

### Introduction

Emotional disorders (ED) (anxiety and mood disorders) are among the most prevalent mental disorders, with a life prevalence of 29 % and comorbidity rates ranging between 40 and 80 % [1, 2]. If the person experiencing the disorder is not adequately treated, the course often becomes chronic and can significantly affect important functioning areas such as work and social relationships [3, 4]. Moreover, the medical care costs and production losses associated with these mental health problems in Europe are huge [2]. These data strongly suggest that efficacious and efficient treatments are needed to address this important health problem [5–8]. Nevertheless, despite these alarming data, evidence exists indicating that most people with depression and anxiety disorders (less than 50 %) do not receive treatment. [9]. To reduce the burden of mental illness, some authors have emphasized the need for an approach that goes beyond the dominant face-to-face treatment approach in order to provide help to people in need of evidence-based treatments, and this approach includes the use of the media, self-help interventions, the use of special settings and information and communication technologies (ICT) [10].

Efficacious psychological treatments for ED currently exist, and a number of evidence-based cognitive-behavioral treatments (CBT) targeting specific disorders have been developed in the past 20 years [11–17]. However, disorder-specific treatment protocols have some problems. First, the high comorbidity rates among ED. Epidemiological studies have shown that at least 55 % of people suffering from depression and an anxiety disorder suffer from another anxiety disorder at the time of the assessment, and this prevalence rate increases to 76 % when different lifespan diagnoses are taken into account [18]. Consequently, clinicians often have to decide on which is the most adequate disorder-specific protocol in these cases, and because these treatments focus on disorder-specific symptomatology, other comorbid diagnoses do not receive sufficient attention [19]. Second, disorder-specific protocols frequently do not target subthreshold symptoms that did not meet diagnostic thresholds for one disorder or another but that may be important to address in the treatment [20]. Third, the high rate in which “not otherwise specified” diagnoses of clinical significance are assigned as current and lifetime conditions for which there are not specific interventions [18]. Finally, the fact that each manualized specific-disorder treatment requires the use of separate handbooks, workbooks and protocols may be an obstacle in the dissemination of evidence-treatments due to its costs and the important amount of training to become adequately familiar with each of the different treatments [20].

### Transdiagnostic approach

In recent years, there has been great interest in treatment strategies (referred to as transdiagnostic treatments) that might be more widely effective across these diverse mental health disorders. Unlike disorder-specific treatment protocols, transdiagnostic treatments generally include treatments aimed at addressing different disorders (for example, different anxiety disorders) with a single protocol [21]. A growing body of research showing the efficacy of transdiagnostic treatments for anxiety disorders [22–27], and for comorbid depression and anxiety disorders [28–30] has emerged in the past years. Moreover, the efficacy and effectiveness of transdiagnostic treatment protocols for ED have been shown in two recent meta-analyses [31, 32].

An important line of research within the transdiagnostic approach is that initiated by D. H. Barlow [20, 33–36]. Barlow’s theory of triple vulnerability emphasizes the underlying vulnerabilities that are common to emotional disorders and help to explain the comorbidity among these diverse conditions [20, 33]. From this theoretical framework, ED are regarded as minor variations in the manifestation of a broader syndrome (that is, “general neurotic syndrome”) such that the development of treatments directly targeting this underlying syndrome rather than symptom-specific variations would result in a more parsimonious, easier to disseminate treatment approach [20]. It would also result in a more inclusive approach, as it lays on the existence of biological and psychological vulnerabilities that are hypothesized to be common among anxiety disorders, unipolar mood disorders, and other disorders such as somatoform and dissociative disorders [20, 37]. Based on this perspective, Barlow’s team designed the Unified Protocol (UP) [37–41], a transdiagnostic treatment protocol that emphasizes the role of emotion regulation in understanding and treating ED. Due to difficulties in emotion regulation, people with ED often react negatively to their own emotions, and they are more likely to use maladaptive emotion regulation strategies that, in turn, increase the frequency and intensity of negative emotions [37]. To enhance adaptive emotion regulation strategies, the UP focuses on four essential aspects: increasing present-focused emotional awareness, addressing emotional avoidance, promoting cognitive flexibility, and facilitating exposure to avoided situations and sensations. The results obtained using this protocol in a traditional face-to-face format demonstrate its effectiveness and are encouraging [30, 38, 42].

The core of all emotion regulation difficulties has been pointed out to be neuroticism/behavioral inhibition (N/BI) [34, 43, 44]. Previous research supports the role of N/BI in accounting for the onset, overlap, and maintenance of ED [33, 44–46]. Literature has also highlighted the role of extraversion/behavioral activation (E/BA) in ED. For instance, structural models have indicated that low E/BA is associated with unipolar depression [47], social anxiety [48] and agoraphobia [49]. Also, a recent meta-analysis indicated that most individuals with anxiety and mood disorders show low levels of E/BA [50]. The effect of the UP on these two temperament dimensions has been demonstrated recently [51].

Literature about Dialectical Behavior Therapy (DBT) has also highlighted the role of emotion dysregulation in psychological disorders [52, 53]. A primary goal in DBT is training patients in adaptive emotion regulation strategies, as emotion dysregulation is assumed to be a key factor in the development and maintenance of these problems [52]. Emotion regulation difficulties have also been shown to be a transdiagnostic factor across a number of psychological disorders, including anxiety and depression [54–58]. A treatment protocol derived from DBT emotion regulation skills training has been tested in a recent study, suggesting that training patients in emotion regulation strategies (for example increasing emotional awareness) may help to reduce anxious and depressive symptoms among distinct ED [59].

### Internet-based treatment protocols

ICT such as the Internet may facilitate access by people for whom traditional therapy is not available [10]. Internet-based treatments have proven to be a very promising tool for solving several mental health problems and enhancing the dissemination of evidence-based treatments [60–63]. Several advantages regarding the recruitment of patients, assessment, diagnosis and case management in Internet-based treatment protocols have been indicated in a recent article [64]. A number of systematic reviews have shown that Internet-based treatments are efficacious [65–69]. Moreover, meta-analyses reveal that these protocols produce higher effect sizes compared to control groups [60, 65, 70] and that they are as efficacious as face-to-face traditional treatments [66, 70–72]. In sum, there is extensive evidence showing the efficacy of these treatments. However, the evidence available about Internet-based treatments is almost exclusively limited to disorder-specific protocols. Indeed, very few studies combining both a transdiagnostic approach and an Internet-based delivery format have been tested through randomized controlled trials (RCT) [25, 26, 29, 73]. Moreover, studies analyzing the efficacy of transdiagnostic Internet-based treatments, address the treatment of anxiety disorders only [25, 26, 73] or have used open-trial designs [28, 74]. Among those focused on anxiety and depression the existing protocols do not contemplate either the treatment of “not otherwise specified” diagnoses or obsessive-compulsive disorder [29], or target a small range of ED [29]. Moreover, to our knowledge, no RCT have been carried out on the effectiveness of a transdiagnostic Internet-based protocol versus treatment as usual (TAU) in public mental specialized care settings.

### Current study

Our research group has developed a traditional transdiagnostic treatment that is partly based on the UP [37]. Taking into account the importance of emotion regulation in the treatment of ED, it also includes components of emotion regulation from DBT [52]. Based on the traditional treatment protocol, we developed EmotionRegulation, an adaptation of this treatment that can be applied online over the Internet.

In this study, we aim to present EmotionRegulation, and test its effectiveness for the treatment of ED in an RCT with a sample made up of participants from specialized care in the Spanish public mental health system, compared to TAU. The ED targeted in this study will be major depression disorder (MDD), dysthymic disorder (DD), panic disorder (PD), agoraphobia (A), social anxiety disorder (SAD), generalized anxiety disorder (GAD), and obsessive-compulsive disorder (OCD). Anxiety disorder not otherwise specified (ADNOS) and (unipolar) mood disorder not otherwise specified (MDNOS) will also be targeted. Secondary objectives will include the following: a) study of the effects of EmotionRegulation on two dimensions of temperament (that is, N/BI and E/BA) and b) study of the acceptability (expectations and opinions) of the online program by patients. In this article, we present the study design.

## Methods/Design

### Study design

**Fig. 1:**
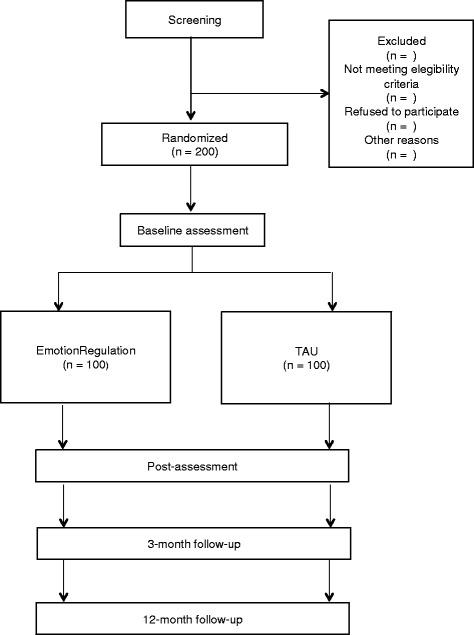
Study flowchart. EmotionRegulation, transdiagnostic Internet-based protocol; TAU, treatment as usual

A two-armed simple-blinded randomized controlled trial will be conducted. Participants will be randomly allocated to one of two conditions: a) EmotionRegulation and b) TAU. Randomization will be stratified by primary diagnosis. Block randomization will be performed within each strata in order to ensure all primary diagnoses are equally represented across conditions. The study will be conducted following the CONSORT statement (Consolidated Standards of Reporting Trials, http://www.consort-statement.org) [75, 76] and CONSORT-EHEALTH guidelines [77]. Participants will be assessed at pre- and post-treatment, and at 3- and 12-month follow-ups. The study flowchart is shown in Fig. 1.

### Study population

The clinical trial will be conducted in the Mental Health Department of the Provincial Consorcio Hospitalario in Castellon and the University Hospital La Ribera in Valencia (Spain). Participants will be adult outpatients from specialized care who attend mental health units to seek psychological and/or psychiatric treatment. Participants will be recruited by clinical psychologists and psychiatrists working in these centers, until the required sample is complete. In order to facilitate the selection of participants in the study, both clinical psychologists and psychiatrists will be given a sheet containing the eligibility criteria.

### Ethics

This trial will be conducted in compliance with the study protocol, the Declaration of Helsinki and good clinical practice. Data security/confidentially will be guaranteed; all relevant EU legislation and international texts on privacy will be observed and respected. Access to the Internet platform is through a unique username-password combination and will be available on a 24/7 basis. All transferred data will be secured via AES-256 encryption.

The study has been approved by the Ethics Committee of University Jaume I (Castellon, Spain) and the Clinical Research Ethics Committee from two hospitals (Consorcio Hospitalario Provincial de Castellon, and Hospital Universitario de la Ribera). The trial was registered at clinicalstrials.gov as NCT02345668. For ethical reasons, patients allocated to TAU will be offered free access to EmotionRegulation after the study has been completed.

### Eligibility criteria

Inclusion criteria will include the following: a) be 18 years or older; b) ability to understand and read Spanish; c) access to Internet at home and having an email address; d) meeting the DSM-IV diagnostic criteria [78] for ED (MDD, DD, MDNOS, PD, A, SAD, GAD, ADNOS, OCD); and e) providing written, informed consent. Exclusion criteria include the following: a) suffering from a severe mental disorder (schizophrenia, bipolar disorder, and alcohol and/or substance dependence disorder); b) the presence of a high risk of suicide; c) medical disease/condition that prevents the participant from carrying out the psychological treatment; or d) receiving another psychological treatment during the study in the experimental group. Receiving pharmacological treatment is not an exclusion criterion during the study period, but patients having an increase and/or change in the medication 2 months prior to enrollment will not be considered for the trial. Also, the increase and/or change in the medication during the study period in the experimental group will imply the participant’s exclusion from subsequent analyses (a decrease in pharmacological treatment is accepted).

### Recruitment

When the psychiatrist or clinical psychologist identifies a potential participant, he or she will describe the study characteristics to him/her. Those candidates interested in participating will sign an informed consent, and the professional will fill out a document describing the participant’s sociodemographic and clinical characteristics, and give him/her a patient information sheet and a handout describing the study. After confirming that the participant has signed the informed consent and understands the study and the treatment options, the researcher will administer assessment instruments related to the inclusion criteria. If the patient fulfills all the study criteria, the researcher will contact an independent researcher to implement randomization. Participants who meet all the inclusion criteria will then be randomized to either EmotionRegulation or TAU and complete the remaining assessment instruments. Participants will be free at any time to withdraw from the treatment or the study without giving any explanation.

### Randomization and blinding

Patients will be randomly allocated in a 1:1 ratio to either of the two groups (EmotionRegulation or TAU) using a computer-generated random number sequence. The Epidat 4.1 program will be used to generate this sequence. The allocation will be carried out by an independent researcher who will be unaware of the characteristics of the study. The sequence will be concealed until interventions are assigned. Patients will agree to participate before the random allocation and without knowing to which treatment they will be allocated. Study researchers conducting psychological assessments (that is, diagnostic interviews) throughout the entire study will be masked to the participants' treatment conditions and unaware of the treatment group to which the patient belongs. For ethical and practical reasons, participants will not be blind to the treatment conditions.

### Interventions

#### Transdiagnostic Internet-based protocol (EmotionRegulation)

Our research group developed a transdiagnostic protocol made up of 12 modules designed for the treatment of the following mental disorders: MDD, DD, MDNOS, PD, A, SAD, GAD, ADNOS and OCD. This protocol is partly based on the UP by David H. Barlow [37] and partly on the emotion regulation skills from DBT by Marsha Linehan [52]. The intervention aims to enhance present-focused emotional awareness, facilitate cognitive flexibility, identify and modify behavioral and emotional avoidance patterns, and promote interoceptive and situational exposure. Each module includes several tasks to practice the different techniques and skills.

We have adapted this protocol for its application on the Internet (EmotionRegulation). EmotionRegulation is an internet-delivered, multimedia, interactive, self-administered program for ED that allows the individuals to learn and practice adaptive ways to regulate their emotions from a transdiagnostic perspective. EmotionRegulation will be delivered through a web platform (https://www.psicologiaytecnologia.com/) designed by our research group. This web platform has four main sections (shown in Table 1).

**Table 1:** Main sections of the web platform (https://www.psicologiaytecnologia.com/)

a) “Home”: This section is the start point from which participants can access the other sections. It also displays a progress bar (0 to 100 %) that shows the progress through the treatment.	
b) “Calendar”: This section shows pending tasks as well as the days in which the participant has accessed the program and has done the module tasks.	
c) “Review”: This section allows participants to review the treatment modules already done as many times as they want.	
d) “How am I?”: This section allows participants to monitor their progress through several graphs as they advance in the program.	

EmotionRegulation includes a Welcome module that provides the participant with general information about the protocol and its objectives, as well as recommendations for benefiting from it, and the following 12 treatment modules:

- M1. Emotional disorders and emotion regulation. This module provides information about the role of emotion regulation in emotional disorders. A brief description of the program modules is also presented, as well as videos with examples of people suffering from different ED.
- M2. Motivation for change. The aims are to analyze the advantages and disadvantages of changing, emphasize the importance of being motivated, and highlight the importance of establishing significant life goals.
- M3. Understanding the role of emotions. This module provides information about the adaptive roles and functions of emotions and the three-component model of emotions.
- M4. The acceptance of emotional experiences. This module aims to teach the patient the acceptance of emotional experiences and its importance in the treatment.
- M5. Practicing acceptance. The objective is to continue to learn about the acceptance of emotional experiences and increase awareness of physical sensations, thoughts, emotions and daily activities.
- M6. Learning to be flexible. It focuses on the importance of maladaptive ways of thinking in the maintenance of emotional disorders, and on learning how to identify them.
- M7. Practicing cognitive flexibility. This module aims to teach the patients the ways maladaptive ways of thinking can be modified. It also provides information about intrusive thoughts and how to deal with them.
- M8. Emotional avoidance. This module aims to teach the patients the emotion avoidance strategies that contribute to the maintenance of emotional disorders.
- M9. Emotion Driven Behaviors (EDBs). The aim is to learn the concept of EDBs, and replace their own maladaptive EDBs with other more adaptive behaviors.
- M10. Accepting and facing physical sensations. The objectives are to teach the patients the role of physical sensations in the emotional response and train them in interoceptive exposure, in order to increase tolerance and promote habituation to physical sensations.
- M11. Facing emotions in the contexts in which they occur. The purpose is the construction of exposure hierarchies to help the patients to begin to face the avoided situations that contribute to the maintenance of the problem.
- M12. Relapse prevention. This module aims to review the strategies learned throughout the program and teach the patient how to identify and cope with future high-risk situations.

These modules are sequential, in order to move through the program step by step. The program duration can vary among the users, and it is estimated that for most participants the duration will be 18 weeks. During the study, EmotionRegulation will be accessible only to participants in the online intervention group. Participants will be allowed to use the program at any time they want during the trial period. See Table 2 for other functionalities in EmotionRegulation.

**Table 2:** Other functionalities in EmotionRegulation

a) Assessments: The program allows the pre-, post- and follow-up instruments to be completed online.	
b) Module self-assessments: Each module ends with a short list of multiple-choice questions that allow participants to assess their understanding of the module and help them to decide whether they need to review its contents.	
c) Automatic e-mails with reminders when participants have not accessed the program in the past 15 days.	
d) Suicide risk alarms: Therapists receive warnings of participants with high risk of suicide (when participants answer questionnaires that include items assessing high suicide risk)	
e) Post-module questionnaires: Each module includes three brief questionnaires (OASIS, ODSIS and PANAS) to evaluate anxiety, depression and positive/negative affect after each treatment module. Participants are able to monitor these scores in the feedback section through the ‘How am I?’ button.	
f) Printable documents: Each module contains several printable documents (PDF) with summaries and self-monitoring sheets that participants are encouraged to use to practice the skills and strategies.	

Participants in the EmotionRegulation condition will be allowed to maintain medication if there are not changes and/or increases but will not be allowed to receive another psychological treatment during the study period. Failure to fulfill these criteria will result in the participant’s data being excluded from data analysis.

#### Treatment as usual

Treatment as usual (TAU) is treatment as delivered in current daily practice by psychiatrists and clinical psychologists in the mental health centers in Spain. TAU may refer to psychiatric treatment, which typically includes prescription and monitoring of antidepressant and/or anxiolytic medication, psychological treatment (this may include case management, group psychotherapy, empathic listening and/or supportive counselling), or a combination of both. Patients in the TAU condition already receiving any of the aforementioned treatments are informed they will continue to receive as usual the services received before enrollment in the study.

### Support

Meta-analyses have shown that attrition rates are higher when no support of any kind is provided to patients in self-administered Internet-based programs [60, 68]. Therefore, we will provide human support and ICT support to all participants in EmotionRegulation.

Human support will be provided by trained pre-doctoral students in our group and will include the following: a) an initial face-to-face session to explain the participant the characteristics of the study and to administer the diagnostic interview to confirm him/her to fulfill the eligibility criteria, b) an initial phone call encouraging participants to start the intervention once baseline assessments have been completed, and c) one weekly brief phone call (maximum of 10 minutes) during the treatment period. The objective of these weekly phone calls will be as follows: 1) to ask the participants about any difficulties or doubts they might have found in the use of the online protocol and help them to solve those problems, 2) to remind them to review the treatment contents as many times as necessary, 3) to emphasize the importance of doing the homework tasks, 4) to encourage participants to keep using the protocol and reinforce them for engaging in the treatment, and 5) to recommend that they complete one module per week. Finally, d) a final phone call will be made after the 18-week treatment period to remind participants that they will be allowed to use the program at any time they want during the trial period and that they will be contacted for follow-up assessments.

ICT support will consist of two weekly mobile phone text messages with reminders about the importance of doing the homework tasks and encouraging participants to review the modules. A commercial platform (www.trendoo.es) will be used to send these messages.

### Instruments

Patients will be assessed at baseline, post-treatment (18 weeks after baseline), and at 3- and 12-month follow-ups. Scores on anxiety, depression and negative and positive affect will also be obtained after each module has been completed. The study variables and assessment times are summarized in Table 3.

**Table 3:** Study variables and assessment points

Instrument	Assessment area	Time of assessment
MINI Neuropsychiatric	Psychiatric diagnosis	Baseline, Post-T and follow-ups
Interview		
BAI	Severity of anxiety	Baseline, Post-T and follow-ups
BDI-II	Severity of depression	Baseline, Post-T and follow-ups
Sociodemographic data	Gender, age, marital status, education, occupation, economic level	Baseline
OASIS	Severity of anxiety	Post-module
ODSIS	Severity of depression	Post-module
SIAS	Severity of SAD symptoms	Baseline, Post-T and follow-ups
PDSS-SR	Severity of PD and agoraphobia symptoms	Baseline, Post-T and follow-ups
PSWQ	Severity of GAD symptoms	Baseline, Post-T and follow-ups
OCI-R	Severity of OCD symptoms	Baseline, Post-T and follow-ups
EQ-5D	Health-related quality of life	Baseline, Post-T and follow-ups
PANAS	Positive and negative affect	Post-module
BIS-BAS	Behavioral inhibition/activation	Baseline, Post-T and follow-ups
ETS	Expectation of treatment	Baseline
OTS	Opinion of treatment	Post-T

Post-T, post-treatment (18 weeks after baseline); follow-ups, 3- and 12-month follow-ups. BAI, Beck Anxiety Inventory; BDI-II, Beck Depression Inventory-II; OASIS, Overall Anxiety Severity and Impairment Scale; ODSIS, Overall Depression Severity and Impairment Scale; SIAS, Social Interaction Anxiety Scale; PDSS-SR, Self-Reported Panic Disorder Severity Scale; PSWQ, Penn State Worry Questionnaire; OCI-R, Obsessive-Compulsive Inventory-Revised; EQ-5D, EuroQoL-5D questionnaire PANAS, Positive and Negative Affect Scale; BIS-BAS, Behavioral Inhibition and Behavioral Activation Scales; ETS, Expectation of Treatment Scale; OTS, Opinion of Treatment Scale

#### Diagnosis interview

Mini International Neuropsychiatric Interview Version 5.0.0 (MINI) [79]. It is a short structured diagnostic psychiatric interview that yields key DSM-IV and ICD-10 diagnoses. The MINI can be administered in a short period of time, and clinical interviewers need only brief training. The MINI has been translated into Spanish and validated [80].

#### Primary outcomes

Beck Anxiety Inventory (BAI) [81]. The BAI is a 21-item self-report measure designed to assess anxiety, with a maximum of 63 points. Each item has a four-point severity scale (for example, not at all, mildly, moderately, and severely) that addresses symptoms experienced during the past week. The internal consistency of the BAI has been found to range from .85 to .94, and it has shown adequate convergent and divergent validity. The Spanish version of the BAI has shown high internal consistency (α = .93) [82].

Beck Depression Inventory (BDI-II) [83]. It is one of the most widely used questionnaires to evaluate depression severity in pharmacological and psychotherapy trials. It consists of 21 items about the different symptoms characterizing major depression disorder, added together to obtain the total score, which can be a maximum of 63 points. The instrument has good internal consistency (α = 0.76 to 0.95). The Spanish version of this instrument has also shown a high internal consistency (α = 0.87) for both the general and clinical populations (α = .89) [84].

#### Secondary outcomes

##### Sociodemographic variables

The following sociodemographic variables will be collected: gender, age, marital status (single, married/relationship, separated/divorced, and widowed), education (years of education), and work status.

##### Diagnosis-specific measures

In order to evaluate the specific anxiety disorder shown by each participant, four different instruments will be implemented. One of the four following questionnaires will be selected and included at pre, post-treatment, and 3- and 12-month follow-up assessments, depending on the main diagnosis given to each participant.

SAD: Social Interaction Anxiety Scale (SIAS) [85]. This scale is made up of twenty items rated from 0 to 4 that assess the anxiety experienced by the patient in social interaction situations. The scale has good internal consistency (α = .88 to .94), good test-retest and discriminant reliability, and appropriate construct validity. The Spanish validation showed adequate internal consistency and good construct validity [86].

PD/A: Self-Reported Panic Disorder Severity Scale (PDSS-SR) [87]. The scale evaluates the severity of the PD symptomatology through measures of panic attack frequency, distress during panic attacks, anticipatory anxiety, fear and agoraphobic avoidance, fear and avoidance of physical sensations, and work and social impairment. Scale reliability (α = .917) and test-retest reliability (ICC = .81) were shown to be excellent. The psychometric analysis of the Spanish version showed excellent internal consistency (α = .85), good test-retest reliability, and adequate convergent validity [88].

GAD: Penn State Worry Questionnaire (PSWQ) [89], which evaluates worry as an uncontrollable, generalized and excessive experience. The PSWQ has good psychometric properties, with an internal consistency ranging from .91 to .95, and good validity and test-retest reliability. The Spanish version of the scale showed an internal consistency of .90 and a test-retest reliability of .82, as well as adequate convergent and discriminant validity [90].

OCD: Obsessive-Compulsive Inventory-Revised (OCI-R) [91]. The OCI-R is a scale made up of 18 items rated from 1 to 4 and organized in six dimensions (washing, verification, order, obsession, hoarding and mental neutralization) that assess obsessive-compulsive behaviors. The OCI-R has showed good internal consistency (α = .81 to .93), good to excellent test-retest reliability (α = .57 to .91) and good convergent validity. The internal consistency of the Spanish version of the OCI-R has been found to be good (α = .86) [92].

##### N/BI and E/BA

Behavioral Inhibition and Behavioral Activation Scales (BIS/BAS) [93]. These scales were designed to assess two temperaments identified in Gray’s biobehavioral theory of emotion [94], namely, behavioral inhibition and behavioral activation. The scale is made up of 20 items rated from 1 to 4, with seven BIS subscale items that evaluate individuals’ emotional responses to impending negative events and 13 BAS items that assess individuals’ behavioral and emotional responses to potentially positive events. The BIS/BAS have demonstrated good reliability in a large sample of individuals with emotional disorders (α = .73 to .92), and stronger associations with other measures of temperament (that is, neuroticism/negative affect and extraversion/positive affect, respectively) than with measures of anxiety or depressive disorder constructs, suggesting that they have good convergent and discriminant validity as indicators of temperament [95]. The internal consistency of the Spanish version ranges between .65 and .82 [96].

##### Post-module measures

Overall Anxiety Severity and Impairment Scale (OASIS) [97]. The OASIS consists of a 5-item questionnaire, rated from 0 to 4, that assesses the frequency and severity of the anxiety symptoms. The instrument also provides measures of avoidance, as well as work, academic, social and everyday life impairment related to anxiety symptoms. A psychometric analysis of the OASIS scale found good internal consistency (α = .80), test-retest reliability (k = .82) and convergent validity for this instrument.

Overall Depression Severity and Impairment Scale (ODSIS) [98]. The ODSIS is a self-report measure with five items that evaluate experiences related to depression. The ODSIS measures the frequency and severity of depression, as well as the level of avoidance, work/school/home interference, and social interference associated with depression. The internal consistency of the scale has been shown to be excellent, with a Cronbach's alpha between .91 and .94 and good convergent and discriminant validity. The Spanish psychometric properties of both the OASIS and the ODSIS are being studied by members of our research team at the time of the publication of this paper.

Positive and Negative Affect Scale (PANAS) [99]. The PANAS consists of 20 items that evaluate two independent dimensions: positive affect (PA) and negative affect (NA). The range for each scale (10 items on each) is from 10 to 50. The Spanish version has demonstrated high internal consistency (α = 0.89 and 0.91 for PA and NA in women, respectively, and α = 0.87 and 0.89 for PA and NA in men, respectively) in college students [100].

##### Quality of life

EuroQoL-5D questionnaire (EQ-5D) [101]. It is a generic instrument that measures health-related quality of life and consists of two parts: Part 1 assesses self-reported problems in each of five domains: mobility, self-care, daily activities, pain/discomfort and anxiety/depression. Each domain is divided into three levels of severity corresponding to no problems, some problems, and extreme problems, yielding a population-based preference score or societal index (SI). A total of 243 theoretically possible health states can be obtained, and the SI is calculated on the basis of these health states. Values range from 1 (best health state) to 0 (death). However, this index may also provide negative values that correspond to health states perceived as worse than death. Utility scores for these health states were assigned using readily available Spanish population tariffs [102]. Part 2 records the subject's self-assessed health on a visual analogical scale (VAS), a 10 cm vertical line on which the best and worst imaginable health states score 100 and 0, respectively.

##### Treatment expectations and treatment opinion

Expectation of Treatment Scale (ETS) and Opinion of Treatment Scale (OTS). These questionnaires are adapted from Borkovec and Nau [103]. The content of the six items, rated on a scale from 0 to 10, cover how logical the treatment seemed, to what extent it could satisfy the patient, whether it could be used to treat other psychological problems, its usefulness for the patient’s specific problem, and to what extent the treatment could be aversive. The expectation scale is applied once the treatment rationale has been explained, at the end of the welcome module. Its aim is to measure subjective patient expectations about this treatment. The opinion scale is administered when the patient has completed the treatment, and its aim is to assess satisfaction with this treatment. Our group has used this questionnaire in several research studies [104, 105].

#### Sample size

The data from an RCT using the UP yielded between-group effect sizes of 0.56 for anxiety and 1.11 for depression, as measured with the BAI and BDI-II, respectively [30]. As we aim to compare the intervention with a TAU group, the results of a meta-analysis comparing CBT transdiagnostic treatments versus TAU have also been considered in the estimation of the expected sample size [106]. This meta-analysis reported a medium post-treatment effect size of 0.44 for depression and of 0.34 for anxiety between transdiagnostic treatment protocols vs. TAU conditions. The type of support we provide in this intervention (contact with researchers before, during and after the treatment period) has also been taking into account when estimating the expected sample size, as defined in a previous meta-analysis focused on Internet-based psychological treatments for depression [107]. Based on a power of .80 in a one-tailed test, an alpha of .05, and an estimated drop-out rate of around 30 % [65, 108] we need a sample size of 100 per condition to detect a post-treatment effect size of 0.40 (Cohen’s d) between both groups. Therefore, the total sample size was determined at 200.

#### Analysis

Intention-to-treat analyses and per protocol analyses will be performed. Reporting of the results will follow CONSORT recommendations [75, 76]. First, the two groups will be compared in order to verify that there are no significant differences between them at baseline using samples t-tests for continuous distributed variables and chi-squares test of independence for categorical to confirm that they are comparable after randomization.

The intention-to-treat principle will be used when analyzing primary and secondary post-treatment data and data collected at the 3- and 12-month follow-ups using mixed effect models with full information maximum likelihood estimation. This method has been recommended for its flexibility over repeated-measures ANOVAs to handle missing date more appropriately [109].

Within and between-group changes will be computed calculating standardized effect sizes (Cohen’s d). Cohen’s d is calculated by dividing the differences between means by the pooled standard deviation [110]. An effect size of 0.20 is considered to be small, of 0.50 to be moderate, and 0.80 and above to be large [110].

Per protocol analyses (compliers only analysis) will also be conducted. Despite this procedure suffers from selection bias, it can help to draw conclusions about the maximum treatment efficacy in patients who comply fully with the treatment [111].

As the trial is still in execution, the state of the art regarding analytic methodology for RCT will be reviewed before analyzing the data, thus variations in the selection of the most appropriate analytic procedures may occur.

## Discussion

This study has several aims. The first is to provide data from a RCT about the effectiveness of a transdiagnostic Internet-based protocol for the treatment of ED in a sample of participants from specialized care in the Spanish public mental health system, compared to TAU. Second, whether the treatment may temper the psychological vulnerability by analyzing its effect on psychological higher-order dimensions (neuroticism/behavioral inhibition and positive affect/behavioral activation) will be studied. The third aim is to study the acceptability of this online program by patients in an ecological setting (public specialized care in Spain).

The advantages of a transdiagnostic Internet-based protocol are two-fold. First, a wide range of ED can be treated with a single protocol, reducing the costs associated with disorder-specific protocols and contributing to solving the problem of comorbidity and NOS diagnoses, as the protocol focuses more on the common pathological processes than on any specific disorder and/or symptomatology. Second, Internet-based protocols can help to disseminate CBT evidence-based treatments, so that more people can benefit from them. This study will provide additional data about the transdiagnostic perspective proposed by Barlow [20], as well as data on the combination of a transdiagnostic perspective and the use of ICTs.

In addition, this study has various strengths. First, this is the first RCT of transdiagnostic Internet-based psychotherapy in specialized care in our country. Positive results achieved with this protocol may have an important impact, since protocols of this type could help to decrease the saturation of the public mental health system, reducing costs and contributing to a general improvement in the public mental health services in our country (for example, reductions in waiting lists, hours of clinical assistance and hours of face-to-face treatment; a higher number of patients who receive psychological treatment; etcetera). Second, the online protocol combines the transdiagnostic cognitive-behavioral principles (psycho-education about emotions, enhancement of cognitive flexibility, interoceptive and situation-based emotion exposure) with components of acceptance and emotion regulation for the treatment of ED. The data obtained with this protocol can help us to understand the psychopathology of these mental disorders. And third, even though transdiagnostic Internet-based protocols are thought to treat different ED, most of the existing studies exclusively target anxiety disorders [25, 26, 73], and others have used open-trial designs [28, 74] and do not contemplate either the diagnosis of obsessive-compulsive disorder or NOS diagnoses [29] or they focus on a smaller number of ED [29]. We consider that this study broadens the current literature about transdiagnostic Internet-based protocols as it is designed for a wide range of anxiety and depressive disorders. Combining the advantages of both a more inclusive transdiagnostic intervention and an Internet-based delivery format may broaden the scope of evidence-based treatments among the population in need. Moreover, the population in which the study is being conducted, that is, patients who attend a variety of public specialized care settings across Spain, can help to draw conclusions about the external validity of the intervention.

Finally, a number of potential limitations should be indicated. First, dropout rates are expected to be high (around 30 %) [67, 110]. Efforts to maintain these drop-out rates below this percentage will be made by providing human support (before, during, and after the intervention) and ICT-support (for example, emails and mobile phone text messages). Second, negative attitudes towards Internet interventions by both clinicians and patients may affect recruitment as well as dropout rates. To minimize the effect of negative attitudes, the nature and characteristics of the intervention will be explained to clinicians involved in the trial. Moreover, for this purpose they will be given a handbook with relevant information about the study (for example objectives of the study, study design, and characteristics of the intervention). In order to increase participant’s credibility, prior to enrollment they will be given a sheet with relevant information concerning the characteristics and objectives of the study, and other issues related to ethics, voluntary participation and confidentiality of the data. Finally, other difficulties could be problems with recruitment, as many people who attend public mental health units do not have access to the Internet at home.

## Trial status

The trial is active and recruiting.

## Abbreviations

A: agoraphobia
ADNOS: anxiety disorder not otherwise specified
BAI: Beck anxiety inventory
BDI-II: Beck depression inventory-II
BIS/BAS: Behavioral Inhibition and Behavioral Activation Scales
CBT: Cognitive Behavioral Treatments
DBT: Dialectical Behavior Therapy
DD: dysthymic disorder
DSM-IV-TR: Diagnostic and statistical manual of mental disorders, fourth text-revised edition
E/BA: Extraversion/Behavioral Activation
ED: emotional disorders
EDB: Emotion driven behaviors
EQ-5D: EuroQoL-5D questionnaire
ETS: Expectation of treatment scale
GAD: generalized anxiety disorder
ICD-10: International classification of diseases 10th revision
ICT: Information and communication technologies
MDD: major depression disorder
MDNOS: mood disorder not otherwise specified
MINI: Mini-international Neuropsychiatric Interview
NA: negative affect
N/BI: neuroticism/behavioral inhibition
NOS: not otherwise specified
OASIS: Overall anxiety and impairment scale
OCD: obsessive-compulsive disorder
OCI-R: obsessive-compulsive inventory-revised
OTS: opinion of treatment scale
ODSIS: Overall Depression and Impairment Scale
PA: positive affect
PANAS: Positive and Negative Affect Schedule
PD: panic disorder
PDSS-SR: Self-reported Panic Disorder Severity Scale
PSWQ: Penn State Worry Questionnaire
RCT: randomized controlled trial
SAD: social anxiety disorder
SI: Societal index
SIAS: Social interaction anxiety inventory
TAU: treatment as usual
UP: unified protocol
VAS: Visual Analog Scale
